# Long‐Range Proton Conduction across Free‐Standing Serum Albumin Mats

**DOI:** 10.1002/adma.201505337

**Published:** 2016-02-03

**Authors:** Nadav Amdursky, Xuhua Wang, Paul Meredith, Donal D. C. Bradley, Molly M. Stevens

**Affiliations:** ^1^ Departments of MaterialsBioengineering and Institute of Biomedical EngineeringImperial College LondonLondonSW7 2AZUK; ^2^ Department of Physics and Centre for Plastic ElectronicsImperial College LondonLondonSW7 2AZUK; ^3^ Centre for Organic Photonics and ElectronicsSchool of Mathematics and PhysicsUniversity of QueenslandBrisbaneQueensland4072Australia

**Keywords:** current–voltage, hopping mechanism, impedance spectroscopy, protein films, proton transfer

## Abstract

**Free‐standing serum‐albumin mats** can transport protons over millimetre length‐scales. The results of photoinduced proton transfer and voltage‐driven proton‐conductivity measurements, together with temperature‐dependent and isotope‐effect studies, suggest that oxo‐amino‐acids of the protein serum albumin play a major role in the translocation of protons via an “over‐the‐barrier” hopping mechanism. The use of proton‐conducting protein mats opens new possibilities for bioelectronic interfaces.

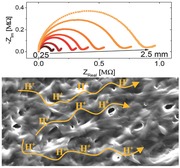

Proton translocation is one of the fundamental processes in nature. The most well‐known examples are proton translocation across the mitochondrial inner membrane or the chloroplast by the adenosine triphosphate synthase complex^1^, ^2^, ^3^ and the proton pump activity of rhodopsin proteins (mainly bacteriorhodopsin protein) in halobacteria.^3^, ^4^, ^5^ Proton transfer (PT) is also essential for numerous biochemical processes such as redox reactions, catalysis, and phosphorylation. In general, protons are being transported by water molecules, although the exact mechanism of PT by bulk water is still debatable.^6^, ^7^ However, protons can be transported laterally along membranes^3^, ^8^, ^9^ or by specific amino acids such as the proton pump activity of bacteriorhodopsin.^3^, ^4^, ^5^

Beyond biological systems, solid‐state proton conductors are commonly used in devices such as batteries and fuel‐cells.^10^, ^11^ In these devices, the most‐used materials are inorganic oxides,^12^ metal‐organic frameworks,^13^ solid acid membranes,^14^ ionic crystals,^15^ and polymeric membranes, where the most common example is Nafion.^16^ Several organic semiconductors have been also shown to have the ability to conduct protons, which enables them to support parallel proton and electron conductivity.^17^, ^18^, ^19^, ^20^ In recent years several bioorganic materials have been also proposed for protonic devices, such as polysaccharide derivatives and melanin pigment.^21^, ^22^, ^23^, ^24^, ^25^ While a wide diversity of materials have been shown to sustain proton current, there are only few types of proton‐conductors: oxide ions, oxoacids (and their anions), and in some cases, heterocycle molecules.^10^ The common denominator for all proton conductors, whether solid‐state materials or water, is the role of a hydrogen bond network that can support long‐range proton conductivity.^6^, ^7^, ^26^

In this study, we explore the use of protein‐based materials as long‐range proton conductors. Due to the abundance of water molecules inside the protein structure and the presence of charged amino acids (mainly oxoacids), proteins are good candidates for the formation of proton conducting materials. Several studies have followed proton conductivity across collagen,^27^ keratin,^28^ and lysozyme layers.^29^, ^30^ Recently, Gorodetsky and co‐workers^31^ showed that upon drop‐casting reflectin (a structural protein found in cephalopods) and drying it between two electrodes, they could measure a proton conductivity across the film of 0.1 mS cm^−1^ at room temperature, and up to 2.6 mS cm^−1^ at 65 °C.

We used bovine serum albumin (BSA), one of the cheapest commercially available proteins, to form an electrospun mat composed of fibrillar structures that can absorb large quantities of water. Our mat is a free‐standing material that can be held and manipulated by hand. First, we explored local (short‐range) proton translocation via excited‐state proton transfer (ESPT) of a photoacid that was absorbed on the mat. Second, we explored long‐range (millimetre length‐scales) proton conductance between two electrodes bridged by the BSA mat with electrochemical impedance spectroscopy (EIS) and current–voltage (*I*–*V*) measurements. We also examined their temperature dependence and the kinetic isotope effect(KIE). We propose that the observed proton translocation is due to an “over‐the‐barrier” proton‐hopping mechanism that involves the oxo‐amino‐acids of the protein as the main proton hopping sites.

It has previously been shown that BSA can be electrospun to form mats.^32^ Following electrospinning, our mats were composed of fibrils with a diameter of several hundred nanometres and large spacing between individual fibrils (**Figure** [qv: **1**]a). After placing them in aqueous solution, the fibrils absorbed water in a sponge‐like manner, and transformed to a thick (>1 μm) fibril surface with almost no spacing between individual fibrils (Figure 1b). By weighing the mats before and after hydration, we could determine the swelling ratio (water content) of the mats to be 143 ± 18% (w/w). The large amount of water within the material distinguishes itself from other bioorganic proton conductors in the literature that contain 5–20% of water.^22^, ^23^, ^24^, ^25^ Following the hydration of the mats, most of the water could be removed by heating or placing the mats in vacuum. By thermogravimetric analysis (TGA), we estimated the amount of the remaining water content of the dehydrated mat to be ≈7% (TGA results are provided as Figure S1 in the Supporting Information). Removal of these water molecules transformed the mat from a flexible film that was easy to hold and manipulate to a highly brittle film. This observation made it clear that water played a major role in the structure of this material. The mats could also be placed in a variety of organic solvents and acids without being dissolved for months (Figure S2, Supporting Information).

**Figure 1:**
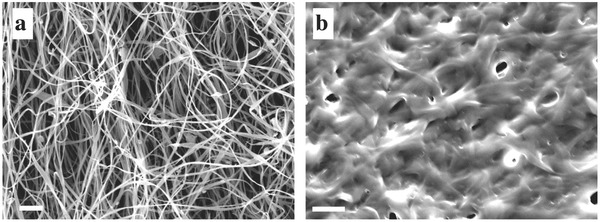
Morphology of the BSA mat. Scanning electron microscopy images of the BSA mats a) before and b) after immersion in water. The scale bar represents 10 μm.

The large amount of water in the BSA mat along with the high ratio of charged amino acids in the structure of the protein (more than one‐third) has encouraged us to explore whether the BSA mat can sustain proton conduction. To probe local PT processes in the BSA mat, we used the photoacid of 8‐hydroxy‐1,3,6‐pyrenetrisulfonate (HPTS, see inset of **Figure** [qv: **2**]a for the protonated, ROH form). Photoacids are molecules with lower p*K*_a_ values in their electronically excited state; for HPTS, an ROH photoacid, the p*K*_a_ decreases from 7.4 in the ground state to 1.3 in its first excited singlet state. Following optical excitation, HPTS in water undergoes an ESPT reaction,^22^, ^23^ as the proton from its hydroxyl group is transferred to the aqueous solvent leaving behind the excited RO^−*^ anion

(1) RO H*⟷k PT ka[RO−*...H+]⇆ diffusion RO−*+H+

**Figure 2:**
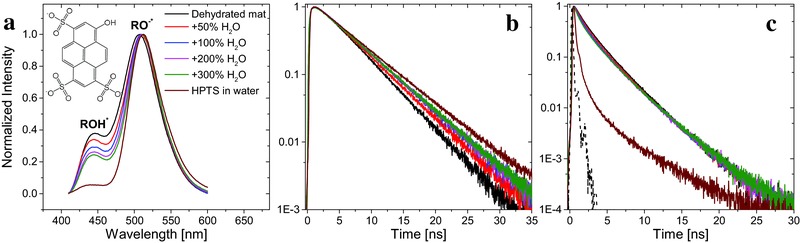
Photo‐induced PT. a) Steady state and b,c) time‐resolved fluorescence of HPTS at the detection wavelength of (b) RO^−*^ and (c) ROH* of the dehydrated mat and with the different weight percentage (%w/w) of added water in comparison to HPTS in water. The dashed line in (c) represents the instrument response function.

After excitation, the proton is transferred to form an ion pair with the deprotonated molecule. The proton can then diffuse to bulk water or recombine with the excited anion to form the ROH* form in what is known as geminate recombination.^33^ Since the ROH* and RO^−*^ forms have different emission wavelengths (for HPTS, 440 and 535 nm, respectively), it is relatively easy to follow their time‐resolved and steady‐state fluorescence.^34^, ^35^, ^36^, ^37^ In pure water, the proton diffuses rapidly from the photoanion, which results in a predominant RO^−*^ species, as can be seen in the steady‐state emission spectra (Figure 2a). On the other hand, when HPTS is in its dry state (powder), the predominant species is the ROH* (Figure S3, Supporting Information), as there is no bulk water in which to diffuse. However, when HPTS was adsorbed on the BSA mat and the mat was dehydrated (leaving ≈7% water content as measured by TGA, Figure S1 in the Supporting Information, and the measurements were conducted under similar room temperature conditions as in the previous experiment in Figure S3, Supporting Information), the predominant form was still RO^−*^. This unexpected finding implies that even in the relatively dry state, HPTS can transfer a proton to nearby molecules. Since there is no excess of water molecules in the dehydrated sample, the proton is likely to be transferred to nearby carboxylates or amines or to the trapped water molecules. Following the gradual addition of small amounts of water to the surface (up to 300% w/w of water), we observed only a slight change in the ROH* band intensity, which was still very different to HPTS in bulk water. Our findings can be explained by a combination of the following explanations: (1) the PT rate of HPTS is slower on the mat than in bulk water; (2) the protons diffuse along the mat in lower dimensionality in comparison to bulk water; and (3) the geminate recombination is more efficient on the mat in comparison to water.

To distinguish between these possibilities, we probed the time‐resolved fluorescence of HPTS at the emission wavelengths of both RO^−*^ (Figure 2b and Figure S4a,b, Supporting Information) and ROH* (Figure 2c and Figure S4c,d, Supporting Information). The PT rate constant (*k*_PT_) can be roughly estimated by: k PT =IRO−F/I ROH F×τRO−−1, where IRO−F/I ROH F is the ratio between the steady‐state intensity of the RO^−*^ and the ROH* bands (Figure 2a) and $\tau_{RO^{-}}$ is the fluorescence lifetime of the RO^−*^ form (Figure 2b). Hence we can estimate *k*_PT_ to be within the range of 5.5 × 10^8^ s^−1^ in the dehydrated mat to 7.6 × 10^8^ s^−1^ in the fully hydrated mat, which is significantly different in comparison to 3.4 × 10^9^ s^−1^ for HPTS in water (Table S1, Supporting Information). The time‐resolved emission of the ROH* form (Figure 2c) can imply the dimensionality of the proton diffusion. In bulk water, protons diffuse in 3D and the ROH* form decays rapidly. However, the decay of the ROH* form is much slower on the BSA mat, even in fully hydrated samples, which might imply that protons diffuse along the BSA mat and not into bulk water. The time‐resolved emission of the ROH* form can be used to get an estimation of the dimensionality of the proton diffusion space, where the fluorescent tail obeys a power‐law of *t* ^−d/2^, where *d* is the diffusion space dimensionality (see further discussion in Supporting Information).^34^, ^36^, ^37^ By plotting a log–log plot (Figure S5, Supporting Information) of the lifetime corrected ROH* decay, we could estimate the fractal space dimensionality of the proton diffusion by linear fitting the first nanoseconds of the decay. We found that for the highly hydrated sample (with 300% w/w water, meaning that this sample contained double the amount of water than the measured swelling ratio of the mat) the dimensionality is 1.07 (and even lower, closer to 1, for the less hydrated samples), compared to 2.99 for HPTS in bulk water (Table S1, Supporting Information), meaning that the protons diffuse along the fibrils within the mats. The role of the geminate recombination of HPTS on the BSA mat is challenging to assess quantitatively. The geminate recombination is interdependent with proton diffusion (Equation (1)), where slower diffusion results in an enhanced geminate recombination.^33^ Proton diffusion along the mat, and especially the dehydrated one, is expected to be slower than water. Indeed, as will be discussed below, proton hopping along the mat is significantly slower in comparison to water.

The ability of HPTS to transfer protons to nearby amino acids has been observed before for the case of HPTS bound in the binding site of natively folded human serum albumin.^38^ It was also recently shown that HPTS can transfer protons to glucosamine units of chitosan.^39^ In order to probe the role of water molecules in the diffusion of the protons, we have followed the KIE of the HPTS time‐resolved measurements (Figure S6, Supporting Information). In water, the KIE of HPTS is ≈3 (Figure S6a, Supporting Information). Surprisingly, we have found no KIE for all the BSA mat samples (Figure S6b–f, Supporting Information), regardless to the percentage of water in the mat. The lack of a KIE implies that proton diffusion in bulk water is not the main proton transfer mechanism in the mat.

In the previous section we showed that within the excited‐state lifetime of HPTS, which limits the measurable transport length of the proton to up to ≈15 nm, the protons in the BSA mat can diffuse along the fibrillar structure of the mat with different kinetics in comparison to water. Next, we investigated whether the mat could serve as an efficient proton conductor in a bioelectronic device. By placing the mat on top of a gold finger electrode structure (Figure S6, Supporting Information), we could examine the distance‐dependent AC electrical impedance response (**Figure** [qv: **3**]a) using EIS and the DC response (Figure 3b) using current voltage (*I*–*V*) measurements. It is important to note that the measurements were conducted in the swollen state of the mat (i.e., at ≈150% (w/w) water). The EIS results, which are represented as a Nyquist plot (the imaginary part of the impedance, *Z*_im_, as the function of the real part, *Z*_real_), exhibit a semi‐circle representative of a parallel RC circuit model (the small spur in the low frequency domain is indicative of charge accumulation in the contacts of through‐plane EIS measurement).^40^ By fitting the semicircle to an RC circuit (Table S2, Supporting Information), we were able to extract the resistance values; these range from 0.10 ± 0.03 to 0.81 ± 0.30 MΩ for *l* = 0.25 and 2.5 mm electrode finger separations, respectively. Taking into account the distance between electrodes, the thickness of the mat (≈75 μm), and the electrode length in contact with the mat (≈7–9 mm), this corresponded to conductivity values of 41.1 and 48.6 μS cm^−1^, respectively (Table S2, Supporting Information). The EIS measurements were complemented with *I*–*V* measurements (Figure 3b) that showed a featureless nonohmic behavior, which is not affected by contact resistance (Figure S7, Supporting Information). Both measurements had a similar distance decay profile (Figure 3c, comparing the measured EIS conductance with the current magnitude in the low bias linear regime of the *I*–*V* curve), with an identical distance‐decay constant (inset of Figure 3c) where the conductance/current is proportional to 1/distance (Figure S8 in the Supporting Information, following Pouillet's law:$\;G\;=\;\sigma \frac{A}{l}$, where *G* is the conductance, *σ* is the conductivity, *A* is the cross‐sectional area (film thickness times electrode length), and *l* is the distance between electrodes), which confirms that the same charge carrier (protons) dictates the conductivity in both AC and DC measurements.

**Figure 3:**
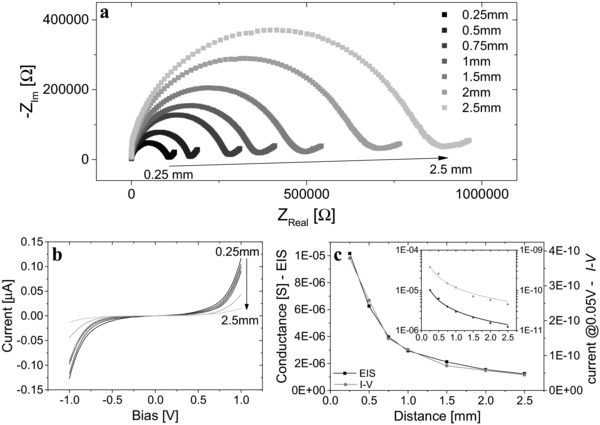
In‐plane proton conductance across BSA mats. a) AC EIS response (plotted on an isometric scale) and b) DC *I*–*V* sweeps for several inter‐electrode separation distances, *l*. c) The distance decay of the EIS conductance (left *y*‐axis, black squares) and the *I*–*V* current at 0.05 V (right *y*‐axis, red squares). The inset shows the same points on a semi‐logarithmic scale spanning two orders of magnitude on both *y*‐axes, along with fits to the same $X^{-0.87}$ dependence.

In order to examine the in‐plane proton conductance mechanism across the BSA mat we measured the KIE and the temperature‐dependence of the conduction process. Similar to the ESPT results (Figure S5, Supporting Information), we found no KIE for the proton conductance for both EIS (**Figure** [qv: **4**]a) and *I*–*V* (Figure S9, Supporting Information) measurements (the KIE of the EIS was slightly inverse (i.e., lower than 1) but within the error range). The EIS temperature dependence studies for two of the measured distances (*l* = 0.75 and 1.5 mm, Figure 4b and Figure S10, Supporting Information, respectively) showed (via an Arrhenius fit) that the proton conductance was thermally activated with *E*_a_ = 0.29 ± 0.02 eV. The temperature dependence of the *I*–*V* measurements showed a similar trend with consistent activation energies for the measured distances (Figure S11, Supporting Information). We note, however, that conductivity measurements (AC and/or DC) on hygroscopic conductors, where the electrical response is strongly affected by the state of hydration, are notoriously unreliable—the temperature dependence can be perturbed or even masked by attendant changes in the hydration state. The fact that in our experiments the water content of the mats was high (≈150% w/w, yielding reduced sensitivity to thermal changes), and further, that we conducted the measurements in a fairly narrow range around room temperature, we consider that the results are moderately robust. In addition, we note that we see an increase in conductance with increasing temperature, whereas were the measurements dominated by dehydration, the opposite would have been expected. Moreover, a simple Arrhenius fit to the temperature dependent *I*–*V* data over this limited range and under circumstances where there will be some change in the state of hydration does not allow us to draw any independent mechanistic conclusions about the underlying transport physics. It does however provide an internally self‐consistent check of the AC data and conclusions.

**Figure 4:**
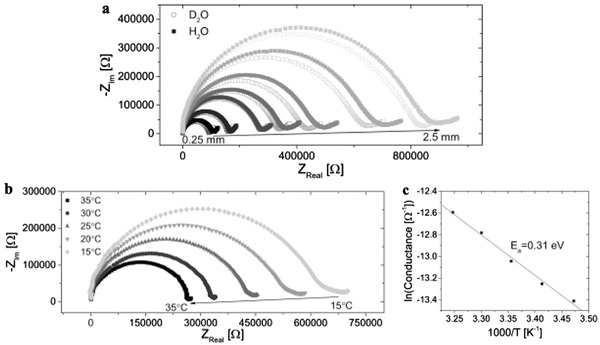
KIE and temperature dependence studies. a) KIE of EIS where water (filled black squares) was replaced with deuterium (open blue squares). b) Temperature dependence of EIS across the *X* = 0.75 mm junction. c) The activation energy of the process in (b) by fitting to an Arrhenius equation ($G\propto \exp(-E_{a}/k_{B}T)$). The graphs in (a) and (b) are displayed on an isometric scale.

Proton conductivity within hydrated samples is commonly explained by the Grotthuss mechanism, which describes proton hopping (diffusion) across water networks, apparently by hopping across the H_9_O_4_^+^ or the H_5_O_2_^+^ cations.^7^ In this mechanism the proton hops from one water molecule to another, and it is expected to have activation energy of 2–3kcal mol^−1^ (≈90–130 meV), and a KIE of 1.4.^7^ Gorodetsky and co‐workers^31^ and Rolandi and co‐workers^23^, ^25^ explained the high proton conductance of dry films of the relectin protein and polysaccharides, respectively, by the Grotthuss mechanism, and also suggested that the protein film contains water channels that support proton conductivity. In the work of Gorodetsky and co‐workers',^31^ similar KIE (≈1.7) values as expected for the Grotthuss mechanism have been found but with a slightly larger *E*_a_ value (≈0.2eV).^31^

In our measurements, we found the following evidences suggesting that the Grotthuss mechanism for proton diffusion across bulk water was not the predominant mechanism for the BSA mat conductivity:

1. The photo‐induced ESPT of HPTS was efficient in the dried sample and its kinetics did not change significantly as water was slowly added to the mat;
2. We found no KIE for both the ESPT kinetics and proton conductance; and
3. The measured activation energy for proton conductance (≈0.29 eV) was higher than expected for water‐mediated proton hopping.

To explain these unique observations, we propose that oxo‐amino‐acids have a significant role as mediators for proton hopping along the BSA mat fibrils since:

1. The ESPT from HPTS to charged amino acids in the mat can be efficient and it should not be affected by the hydration level of the mat;
2. The protons of the charged amino acids in the mat are not expected to be deuterated by the addition of D_2_O, so the KIE should be 1 as observed; and
3. The activation energy in the Grotthuss mechanism is related to the hydrogen bond strength in water.^7^ The larger hydrogen bond strengths between carboxylates and between carboxylates and amines^26^ might then explain a higher activation energy.

Though it is hard to visualize or predict how exactly the network of oxo‐amino‐acids and trapped water molecules are arranged in space to support the proton translocation, it is very likely that the proton conductivity mechanism within the BSA mat can be related to lateral proton diffusion as observed next to membrane–water interfaces,^8^, ^9^ and even to some extent in BSA monolayers at a water–air interface.^41^ This mechanism is also one of the proposed mechanisms to justify the high proton conductivity for Nafion films, where a water supported network of sulfonic acid moieties might serve as the proton hopping sites,^42^ though it is important to note that the conductivity of Nafion is orders of magnitude higher than measured here for BSA mats.

We further attempted to fit our experimental results to the theoretical paradigm of proton hopping to estimate the hopping rate and the distribution of hopping sites in the mat. Proton‐hopping can be envisioned as a small polaron problem where proton donors and acceptors are bridged by a hydrogen bond; each hopping event corresponds to a phonon‐assisted tunneling across a barrier.^43^ In general, an under‐the‐barrier event can be distinguished from an over‐the‐barrier one (Figure S12a, Supporting Information, for schematic representation). The activation energy (*E*_a_) in an under‐the‐barrier event is of the order of *ℏ*ω (where *ω* is the optical phonon frequency and *ℏ* is the reduced Planck constant), and the PT rate, *k*_PT_, can be expressed by the common expression for a nonadiabatic tunneling event.^43^ For an over‐the‐barrier event, the proton resides on the bridge (with a lifetime of $\;\tau_{\mathrm{PT}}$)^44^ and the activation energy serves as the barrier where $E_{a}>\hbar \omega$.^43^ For the same donor and acceptor, an over‐the‐barrier event will have larger *E*_a_ and will be the dominant mechanism at higher temperatures (Figures S11b and S12b for schematic representation, Supporting Information). Our measured activation energy (≈0.29 eV) in comparison to the optical phonon frequency (estimated by infrared (IR) spectroscopy (Figure S13, Supporting Information) to be ≈3300 cm^−1^ i.e., with *ℏ* *ω* = 65 meV) is suggestive of over‐the‐barrier proton hopping. In this case, τ_PT_ can be expressed as

(2) τ PT −1=ωℏ exp −EakBT

where *k*_B_ is the Boltzmann constant and *T* is temperature. Using the measured values for activation energy and phonon frequency, we estimate τ PT −1 to be ≈1 × 10^10^ s^−1^ (τ_PT_≈100 ps), a significantly higher value than the PT rate in water (≈1.5 ps), which is considered an under the barrier PT.

We can further fit our measured conductivity values using the Nernst–Einstein relation between conductivity (*σ*) and charge carrier mobility (*μ*)

(3) $$\begin{array}{c} \sigma \;=\;ne\mu \end{array}$$

where *n* is the charge density and *e* the electron charge. The charge carrier mobility is further proportional to the diffusivity of the charge carrier

(4) $$\begin{array}{c} \mu \;=\frac{e}{k_{B}T}\;D \end{array}$$

with diffusion constant

(5) D=τ PT −1r nn 2

where *r*_nn_ is the average hopping distance. Combining Equations (3)–(5) yields the following relation for the mat conductivity

(6) σT=ne2r nn 2τ PT −1kBT

The distance between proton donor and acceptor (*r*_nn_) for a single hopping event is determined by the length of the hydrogen bond and it is confined to a narrow range around 2.5 Å. Using the above calculated PT lifetime (≈100 ps), we can estimate the charge carrier density to be 1 × 10^18^ cm^−3^ (=1.7 × 10^−6^ mol cm^−3^ = 1.7 m), which can be compared to the concentration of hydronium ion at pH 2.8. The 2.5 Å hopping distance was chosen for cases where the proton donor and acceptor are bridged by a hydrogen bond, as discussed for the Grotthuss mechanism.^7^ However, according to the Eigen–Weller model, a PT event can be also mediated by up to two water molecules, which leads to an optimal PT distance of ≈7 Å.^45^ On using the latter value for *r*_nn_, the estimated charge carrier density in the mat is reduced to 1.5 × 10^17^ cm^−3^. It is also important to note that the measured conductivity values may be underestimated as they consider the entire cross‐section of the mat in calculations, while the charges most likely migrate along a narrower cross‐section closer to the electrodes and along specific pathways of the fibril structure.

In summary, we followed the in‐plane PT within free‐standing BSA mats by photo‐induced ESPT, EIS, and *I*–*V* measurements. We found that protons could be transferred along the fibrillar structure of the mat which allows long‐range (millimetre length‐scales) proton transport with one of the highest deduced conductivity values (≈50 μS cm^−1^) for biological materials. Temperature dependence and KIE measurements suggest that oxo‐amino‐acids have a significant role in the PT mechanism. Our results support an over‐the‐barrier proton‐hopping mechanism and we were able to calculate the proton lifetime on the bridge (barrier) to be in the order of 100 ps. Together with the Nernst–Einstein relation, we used this lifetime to fit our measured conductivity values and estimate the charge carrier density of the hopping sites in the mat to be 2 × 10^18^ cm^−3^.

Using proteins as materials for proton conduction opens new and exciting possibilities for bioelectronic devices. BSA mats are biocompatible and can support cell proliferation,^46^ which makes this system ideal for the study of proton translocation between cells. Moreover, BSA mats are highly robust and can be placed in many organic solvents and acids without being dissolved. Together with their high proton conductivity, BSA mats could be easily used in proton conducting devices such as fuel cells and batteries. BSA mats distinguish themselves from other bioorganic proton conductors, and also from common inorganic conductors, as they are extremely cheap (in the order of 1 GBP g^−1^) and can be easily processed to form electrospun mats in the metre length‐scale.

## Supporting information

As a service to our authors and readers, this journal provides supporting information supplied by the authors. Such materials are peer reviewed and may be re‐organized for online delivery, but are not copy‐edited or typeset. Technical support issues arising from supporting information (other than missing files) should be addressed to the authors.

Supplementary

## References

[1] J. Weber , A. E. Senior , FEBS Lett. 2003, 545, 61. 12788493 10.1016/s0014-5793(03)00394-6

[2] P. D. Boyer , Annu. Rev. Biochem. 1997, 66, 717. 9242922 10.1146/annurev.biochem.66.1.717

[3] R. J. P. Williams , Annu. Rev. Biophys. 1988, 17, 71. 10.1146/annurev.bb.17.060188.000443 2840089

[4] J. Heberle , Biochim. Biophys. Acta, Bioenerg. 2000, 1458, 135. 10.1016/s0005-2728(00)00064-5 10812029

[5] H. Luecke , H. T. Richter , J. K. Lanyi , Science 1998, 280, 1934. 9632391 10.1126/science.280.5371.1934

[6] C. Knight , G. A. Voth , Acc. Chem. Res. 2012, 45, 101. 21859071 10.1021/ar200140h

[7] N. Agmon , Chem. Phys. Lett. 1995, 244, 456.

[8] M. G. Wolf , H. Grubmüller , G. Groenhof , Biophys. J. 2014, 107, 76. 24988343 10.1016/j.bpj.2014.04.062 PMC4119267

[9] C. Zhang , D. G. Knyazev , Y. A. Vereshaga , E. Ippoliti , T. H. Nguyen , P. Carloni , P. Pohl , Proc. Natl. Acad. Sci. USA 2012, 109, 9744. 22675120 10.1073/pnas.1121227109 PMC3382531

[10] K. D. Kreuer , S. J. Paddison , E. Spohr , M. Schuster , Chem. Rev. 2004, 104, 4637. 15669165 10.1021/cr020715f

[11] K. D. Kreuer , Chem. Mater. 1996, 8, 610.

[12] K. D. Kreuer , Annu. Rev. Mater. Res. 2003, 33, 333.

[13] P. Ramaswamy , N. E. Wong , G. K. H. Shimizu , Chem. Soc. Rev. 2014, 43, 5913. 24733639 10.1039/c4cs00093e

[14] S. M. Haile , C. R. I. Chisholm , K. Sasaki , D. A. Boysen , T. Uda , Faraday Discuss. 2007, 134, 17. 17326560 10.1039/b604311a

[15] S. Horike , D. Umeyama , M. Inukai , T. Itakura , S. Kitagawa , J. Am. Chem. Soc. 2012, 134, 7612. 22512400 10.1021/ja301875x

[16] K. D. Kreuer , J. Membr. Sci. 2001, 185, 29.

[17] E. Stavrinidou , O. Winther‐Jensen , B. S. Shekibi , V. Armel , J. Rivnay , E. Ismailova , S. Sanaur , G. G. Malliaras , B. Winther‐Jensen , Phys. Chem. Chem. Phys. 2014, 16, 2275. 24352071 10.1039/c3cp54061h

[18] E. Stavrinidou , P. Leleux , H. Rajaona , D. Khodagholy , J. Rivnay , M. Lindau , S. Sanaur , G. G. Malliaras , Adv. Mater. 2013, 25, 4488. 23784809 10.1002/adma.201301240

[19] L. Herlogsson , X. Crispin , N. D. Robinson , M. Sandberg , O. J. Hagel , G. Gustafsson , M. Berggren , Adv. Mater. 2007, 19, 97.

[20] K. Tybrandt , K. C. Larsson , A. Richter‐Dahlfors , M. Berggren , Proc. Natl. Acad. Sci. USA 2010, 107, 9929. 20479274 10.1073/pnas.0913911107 PMC2890459

[21] Y. Deng , B. A. Helms , M. Rolandi , J. Polym. Sci., Part A: Polym. Chem. 2015, 53, 211.

[22] J. Wuensche , Y. Deng , P. Kumar , E. Di Mauro , E. Josberger , J. Sayago , A. Pezzella , F. Soavi , F. Cicoira , M. Rolandi , C. Santato , Chem. Mater. 2015, 27, 436.

[23] C. Zhong , Y. Deng , A. F. Roudsari , A. Kapetanovic , M. P. Anantram , M. Rolandi , Nat. Commun. 2011, 2. 10.1038/ncomms1489 21934660

[24] A. B. Mostert , B. J. Powell , F. L. Pratt , G. R. Hanson , T. Sarna , I. R. Gentle , P. Meredith , Proc. Natl. Acad. Sci. USA 2012, 109, 8943. 22615355 10.1073/pnas.1119948109 PMC3384144

[25] Y. Deng , E. Josberger , J. Jin , A. F. Roudsari , B. A. Helms , C. Zhong , M. P. Anantram , M. Rolandi , Sci. Rep. 2013, 3, 2481. 24089083 10.1038/srep02481 PMC3789148

[26] T. Steiner , Angew. Chem. Int. Ed. 2002, 41, 48.

[27] G. H. Bardelmeyer , Biopolymers 1973, 12, 2289. 4757325 10.1002/bip.1973.360121008

[28] E. J. Murphy , J. Colloid Interface Sci. 1976, 54, 400.

[29] R. H. Tredgold , R. C. Sproule , J. McCanny , J. Chem. Soc., Faraday Trans. 1 1976, 72, 509.

[30] G. Careri , M. Geraci , A. Giansanti , J. A. Rupley , Proc. Natl. Acad. Sci. USA 1985, 82, 5342. 3860864 10.1073/pnas.82.16.5342 PMC390564

[31] D. D. Ordinario , L. Phan , W. G. Walkup Iv , J.‐M. Jocson , E. Karshalev , N. Hüsken , A. A. Gorodetsky , Nat. Chem. 2014, 6, 596. 24950329 10.1038/nchem.1960

[32] Y. Dror , T. Ziv , V. Makarov , H. Wolf , A. Admon , E. Zussman , Biomacromolecules 2008, 9, 2749. 18803419 10.1021/bm8005243

[33] E. Pines , D. Huppert , N. Agmon , J. Chem. Phys. 1988, 88, 5620.

[34] N. Amdursky , R. Simkovitch , D. Huppert , J. Phys. Chem. B 2014, 118, 13859. 25380297 10.1021/jp509153r

[35] L. M. Tolbert , K. M. Solntsev , Acc. Chem. Res. 2002, 35, 19. 11790085 10.1021/ar990109f

[36] P. Leiderman , L. Genosar , D. Huppert , J. Phys. Chem. A 2005, 109, 5965. 16833931 10.1021/jp050037b

[37] D. B. Spry , A. Goun , K. Glusac , D. E. Moilanen , M. D. Fayer , J. Am. Chem. Soc. 2007, 129, 8122. 17567012 10.1021/ja071939o

[38] B. Cohen , C. M. Alvarez , N. A. Carmona , J. A. Organero , A. Douhal , J. Phys. Chem. B 2011, 115, 7637. 21476505 10.1021/jp200294q

[39] R. Simkovitch , D. Huppert , J. Phys. Chem. A 2015, 119, 641. 25556376 10.1021/jp511349j

[40] T. Soboleva , Z. Xie , Z. Shi , E. Tsang , T. Navessin , S. Holdcroft , J. Electroanal. Chem. 2008, 622, 145.

[41] B. Gabriel , J. Teissié , Proc. Natl. Acad. Sci. USA 1996, 93, 14521. 8962084 10.1073/pnas.93.25.14521 PMC26165

[42] P. Choi , N. H. Jalani , R. Datta , J. Electrochem. Soc. 2005, 152, E123.

[43] H. Böttger , V. V. Bryksin , Hopping Conduction in Solids, VCH, Weinheim 1985.

[44] It is important to distinguish between the proton transfer rate and lifetime of the hopping events as discussed in this section to the proton transfer rate of HPTS as discussed above.

[45] M. Rini , B.‐Z. Magnes , E. Pines , E. T. J. Nibbering , Science 2003, 301, 349. 12869756 10.1126/science.1085762

[46] S. Fleischer , A. Shapira , O. Regev , N. Nseir , E. Zussman , T. Dvir , Biotechnol. Bioeng. 2014, 111, 1246. 24420414 10.1002/bit.25185

