# Supplementary material for: Long‐Range Proton Conduction across Free‐Standing Serum Albumin Mats
Source: Adv Mater. 2016 Feb 3;28(14):2692–8. doi: 10.1002/adma.201505337 (PMC4862025; doi:10.1002/adma.201505337)
Supplement: Supplementary file 1 — Supplementary [file ADMA-28-2692-s001.pdf]

Copyright WILEY-VCH Verlag GmbH & Co. KGaA, 69469 Weinheim, Germany, 2016.

ADVANCED MATERIALS

Supporting Information

for *Adv. Mater.*, DOI: 10.1002/adma. 201505337

Long-Range Proton Conduction across Free-Standing Serum Albumin Mats

Nadav Amdursky, Xuhua Wang, Paul Meredith, Donal D. C. Bradley, and Molly M. Stevens*

Supporting Information

Long-Range Proton Conduction Across Free-Standing Serum Albumin Mats

Nadav Amdursky, Xuhua Wang, Paul Meredith, Donal D. C. Bradley, Molly M. Stevens**

Materials and Methods

Electrospinning of BSA mat **–** BSA (Sigma-Aldrich) was used to form the mats according to the protocol of Fleischer et al.[1] BSA was dissolved in 90% TFE to a final concentration of 14% (w/v). β-mercaptoethanol was added to the solution to a final concentration of 5% (v/v). A custom-built electrospinning system was used, where a bias of 11.5kV was applied on an 18-gauge blunt needle, while the collector was grounded. The distance between the collector and the end of the needle was ~11cm, and the rate of injection was 0.9 mL/min.

Scanning electron microscopy (SEM) characterisation **–** small pieces from the mat were coated with a thin layer of Au, and imaged with a SEM JEOL 5610LV system, at an operating bias of 17-20kV.

Steady-state and time-resolved fluorescence **–** Dry BSA mat was placed in a 0.05mM solution of HPTS for ~4h. The hydrated mat with HPTS was placed *in vacuo* over night for complete dehydration. Deionised water was added to the mat in small aliquots to reach the desired water percentage. The dehydrated mat was weighed before the experiment in order to calculate the desired amount of water. A Fluorolog system (Horiba) with 1 nm bandpass slits in both the entrance and exit arms was used for the steady-state measurements and the sample was excited at 390nm. A Deltaflex system (Horiba) was used for time-resolved emission spectroscopy with a 405nm laser diode (<100ps pulse duration) as excitation source and data (at least 20,000 counts) were collected at 440 and 530nm.

Finger electrode preparation **–** A custom stainless steel shadow mask was fabricated with the desired finger electrode pattern. Freshly-cleaned microscope slides were used as substrates

onto which a 100nm thickness gold pattern was evaporated through the shadow mask using a MBraun thermal evaporator (5 x 10^{-7}mbar) inside a nitrogen filled overpressure glovebox system. Prior to gold deposition, a 10nm thickness chromium layer was first evaporated to act as an adhesion promotion for the gold.

Impedance Spectroscopy – The impedance measurements were carried out using an SI 1260 impedance/gain-phase analyser (Schlumberger). At least 24h before the measurement the dry mats were placed in deionised water. The wet mats were then placed onto the gold finger electrode substrates (Fig. S6) and were dried with filter paper to remove excess water not tightly bound to the surface. Micromanipulator probes were used to contact the gold electrodes. A frequency range of 10MHz – 100Hz was used with applied a.c. bias = 100mV and integration time = 0.5s; no d.c. bias was applied. The impedance spectra were fitted using ZView software (© Scribner Associates, Inc), allowing extraction of the resistance and capacitance values for each junction.

Current-Voltage measurements – The current-voltage sweeps were carried out using a Keithley 2400 source-measure unit controlled by computer via a home written Labview program. The same electrode format, BSA sample preparation and micromanipulator probe set ups were used for these d.c. experiments except that one of the probes was biased while the other was grounded. The scan rate was 20mV/s in the ±1V bias range with a voltage step of 0.01V. The presented *I-V* curves are averages of forward (-1V→+1V) and reverse (+1V→-1V) scans. The conductance/resistance values were extracted from these current-voltage measurements by linearly fitting the low bias (±0.05V) region.

KIE measurements – The dry electrospun mat was placed in deuterium oxide (Sigma-Aldrich >99.9%) for at least 24h before measurement. For the time-resolved measurements, the mat was then dehydrated in vacuum overnight, and the measurements were conducted by adding small amounts of D_2O to reach the desired deuterium percentage. The subsequent sample preparation for EIS and I-V measurements was exactly as for non-deuterated samples.

Temperature dependence **–** The finger pattern electrodes with BSA mats on top were placed on a heater that was controlled by a Keithley temperature controller. The sample was equilibrated for ~5min at each temperature point (starting from 15ºC and rising to 35ºC) and the EIS and *I-V* measurements were performed as already discussed above.

Fourier Transform Infrared (FTIR) measurements **–** FTIR measurements used a Perkin Elmer Spectrum 100 FTIR spectrometer with a single bounce Ge-ATR at 16°C. A background scan was measured before the sample. The spectrum was collected in the range 900-4000cm^{-1}, with a 1cm^{-1} increment. Both background and sample measurements were averaged over 10 scans.

TGA measurements **–** A disc (~6 mm diameter) of the BSA mat was placed in water for several hours following by de-hydration in vacuum over-night. The TGA measurement of the de-hydrated mat was conducted with a Netzsch STA449 F5 Simultaneous DSC/TGA.

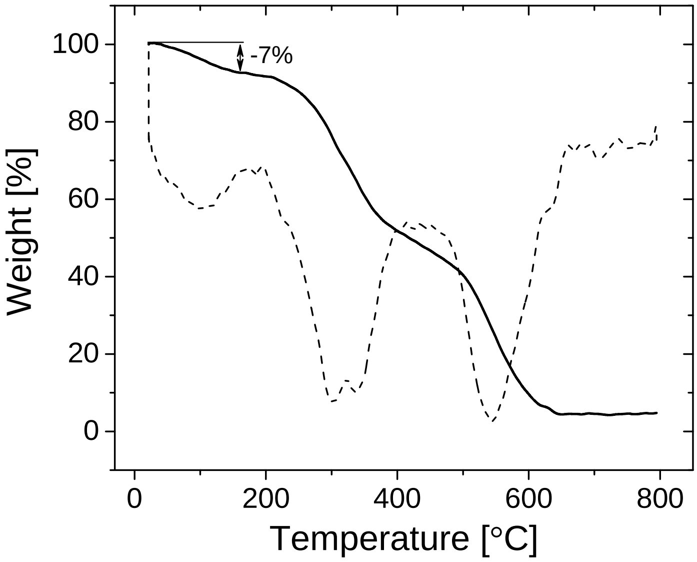

Figure S1. Thermogravimetric analysis of the dehydrated BSA mat. The dashed line is the derivative of the weight loss profile.

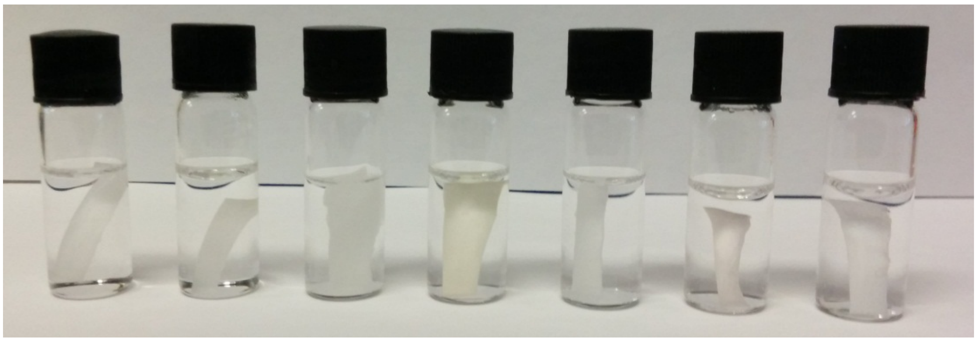

Figure S2. BSA mats in various solvents. The mats were placed for 3 months in (from left to right): Chloroform, Dimethyl sulfoxide, 1,1,1,3,3,3-Hexafluoro-2-propanol, Acetone, 2,2,2-Trifluoroethanol, 1M HCl and Deionised water.

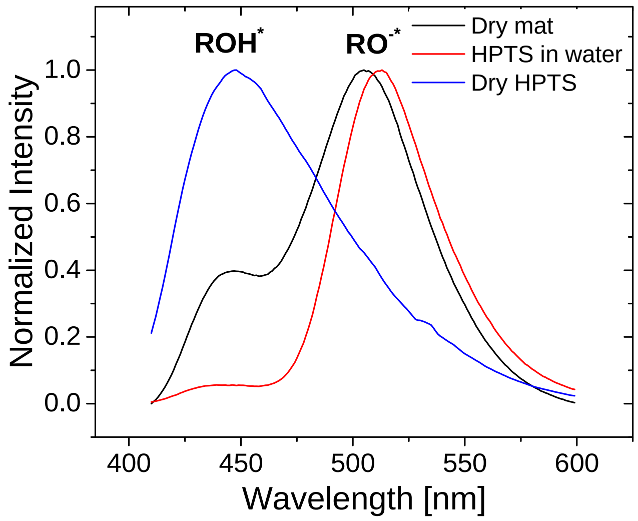

Figure S3. Steady state fluorescence spectrum of HPTS in dehydrated BSA mat, in bulk water and in dry condition (powder on glass).

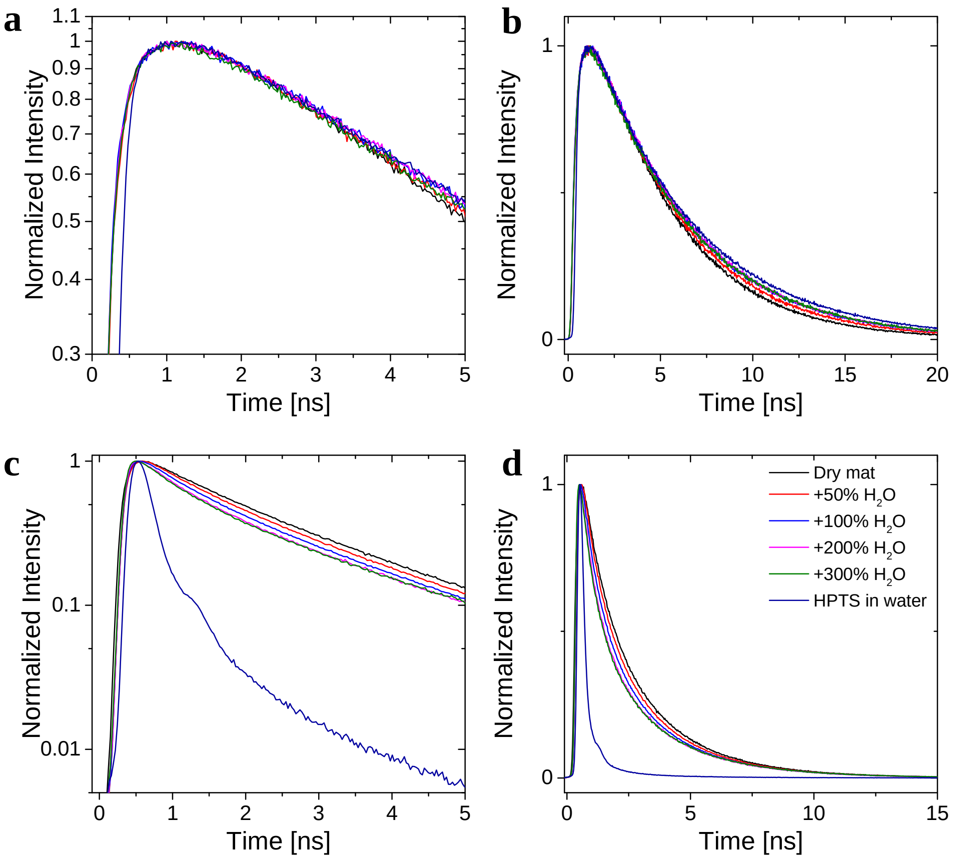

Figure S4. Time-resolved spectra of HPTS on BSA mats. (a) Zoom area and (b) linear scale of Fig. 2b in the main text. (c) Zoom area and (d) linear scale of Fig. 2c in the main text.

Table S1. Calculated fractal space dimensionality of the proton diffusion.

Sample	$I^F_{RO^-}/I^F_{ROH}$	τ_{RO^-} (ns)	k_{PT} (s^{-1})	Calculated power-law factor[a]	Derived space dimensionality
Dehydrated BSA mat	2.6	4.9	5.6×10^8	0.500 (0.970)	1.00
+50% H_2O	2.9	5.1	5.9×10^8	0.510 (0.976)	1.02
+100% H_2O	3.4	5.4	6.4×10^8	0.530 (0.980)	1.06
+200% H_2O	3.8	5.4	7.1×10^8	0.530 (0.987)	1.06
+300% H_2O	4.1	5.4	7.6×10^8	0.535 (0.990)	1.07
HPTS in bulk H_2O	18.5	5.5	3.4×10^9	1.495 (0.994)	2.99

[a]The brackets display the adjusted R^2 of the fit.

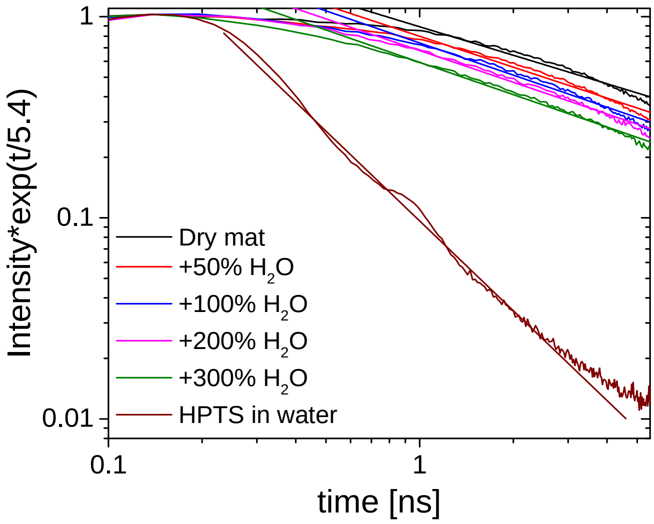

Figure S5. Log-log plot of the time-resolved kinetics, after multiplying by exp(t/5.4), where 5.4 is the radiative life time in ns, together with the fit that was used to extract the power law factor (Table S1).

Fractal space dimensionality of the proton diffusion

The ROH^* decay has a non-exponential long-time fluorescence tail with a power-law decay of $t^{-\alpha}$. Due to the high fluorescence intensity of HPTS, one can follow the tail intensity over a long time scale (into the nanosecond regime). The decay of the ROH^* fluorescence is very sensitive to the space dimensionality of the proton diffusion. In back-to-back publications, Huppert, Agmon and Pines developed the numerical solution for HPTS (or any other photoacid) kinetics.[2,3] They showed that if the ROH* fluorescent decay (I_f^{ROH}) is multiplied

by exp[t/τ_r], where τ_r (=5.4 ns) is the radiative decay lifetime, the factor (α) of the power-law is proportional to d/2, where the d is the space diomensionality of the proton diffusion:

$$I_f^{ROH}(t)\exp[t/\tau_r] \sim \frac{\pi a^2 k_a \exp[-V(a)]}{2k_{PT}(\pi D)^{d/2}} t^{-d/2}$$

where D is the proton diffusion constant, k_a is the proton geminate recombination rate constant, k_{PT} is the proton transfer rate constant, and a is the radius of the reaction sphere, where the reaction sphere potential, V(a), is proportional to R_D/a, where R_D is the Debye radius. R_D is expressed as:

$$R_D = \frac{|z|e^2}{\varepsilon k_B T}$$

where z is the RO^- form charge (in electron charge units), e is the electron charge, ε is the dielectric constant of the medium and k_B is the Boltzmann constant.

For HPTS in bulk water the protons diffuse in 3-D from the excited HPTS molecule, which means that the power-law factor is 3/2 (d=3). In Table S1 we summarize the calculated space dimensionality of the proton diffusion in each mat sample containing different amounts of water, in comparison to HPTS in bulk water. It is mportant to stress that this approximation is an analytical solution based on the ESPT of photoacids (Eq.(1) in the main text). However, in some cases, the experimental results cannot be well fitted using this analytical solution (for instance, the decay of HPTS in the dehydrated mat), and the extracted value of dimensionality should, therefore, be considered with great care.

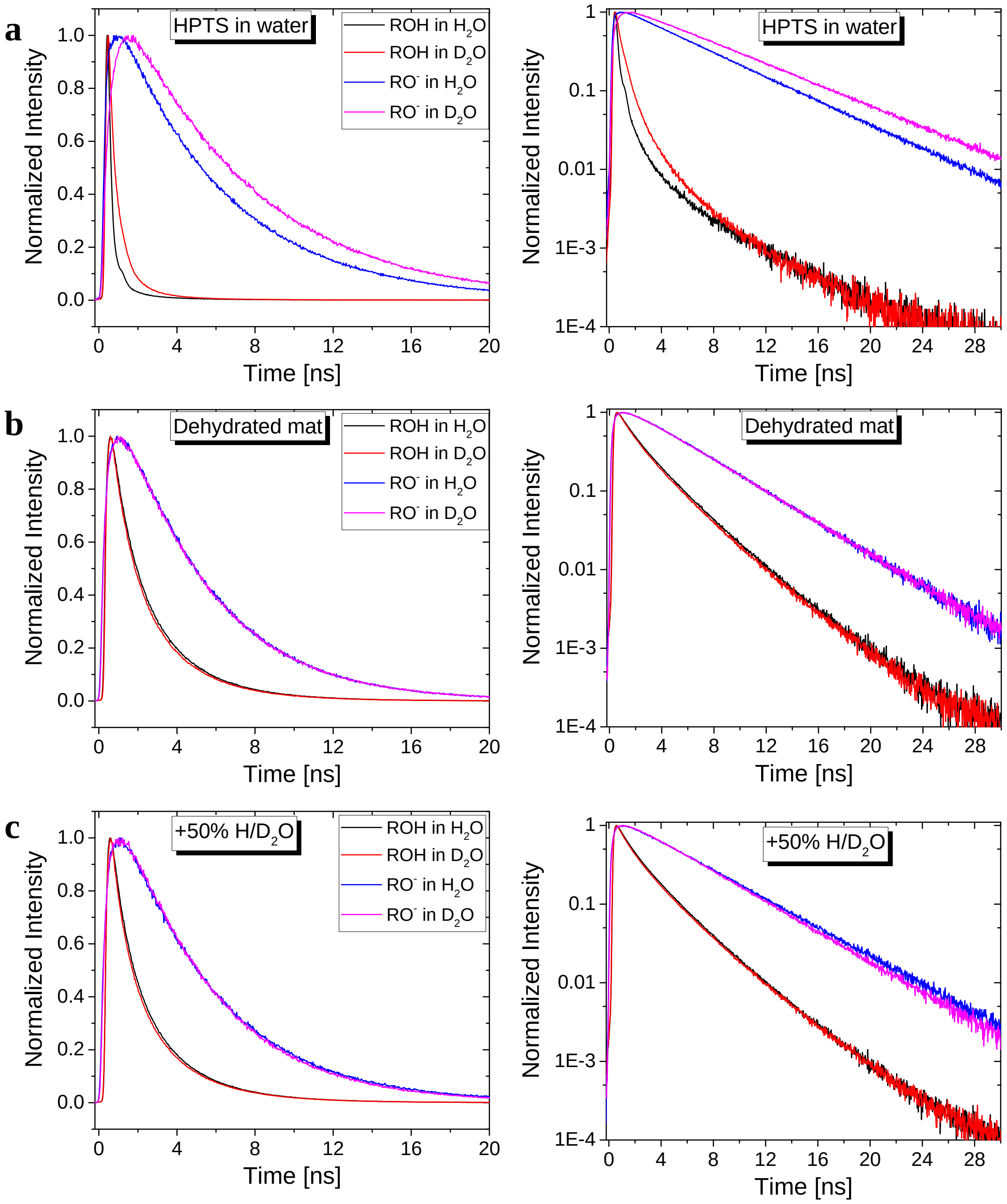
a
HPTS in water
ROH in H2O
ROH in D2O
RO- in H2O
RO- in D2O
Normalized Intensity
Time [ns]
b
Dehydrated mat
c
+50% H/D2O

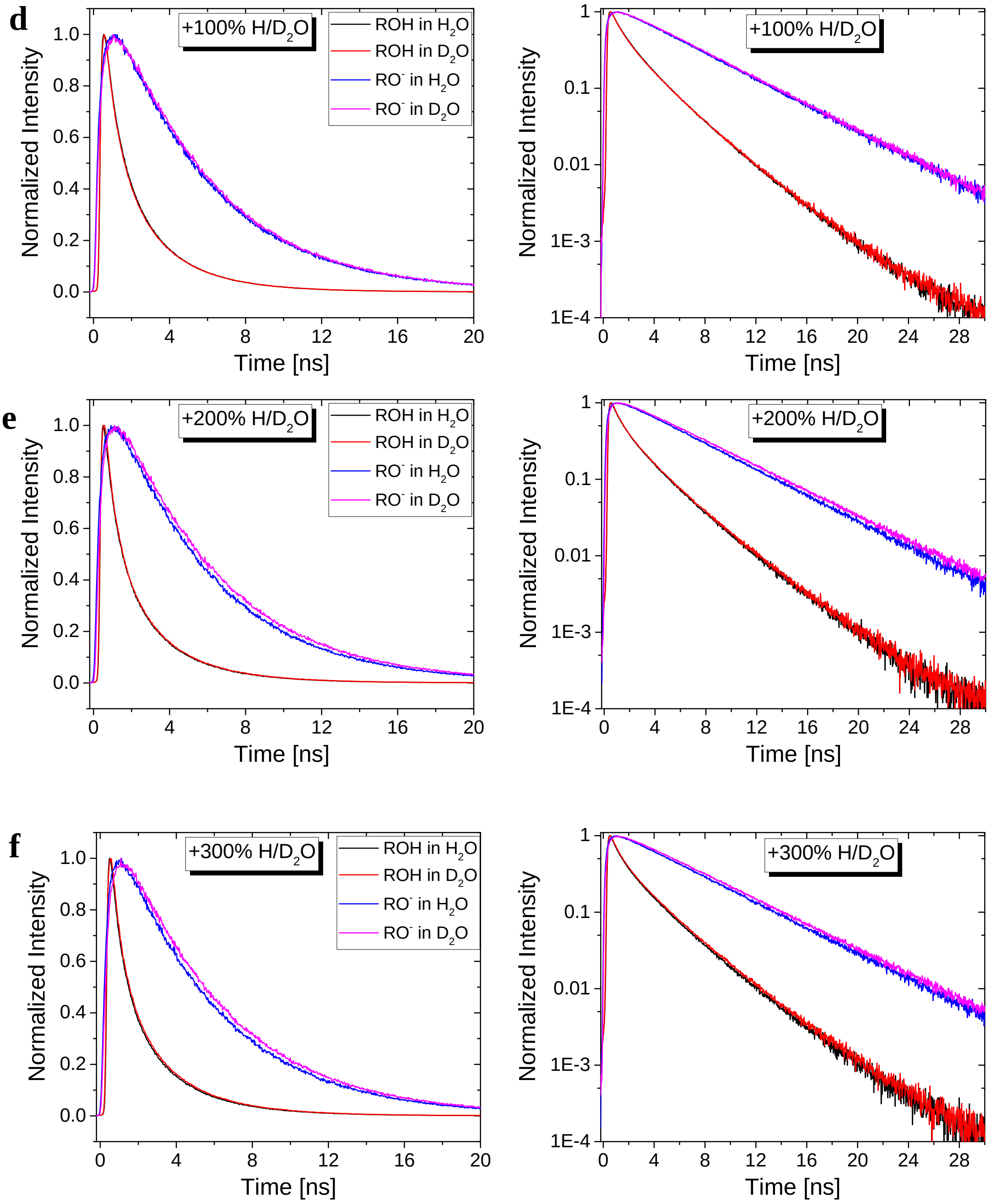

Figure S6. KIE of HPTS kinetics of (a) HPTS in water, (b) in the dehydrated mat and upon the addition of (c) 50%, (d) 100%, (e) 200% and (f) 300% of D_2O. The left graphs present the data on a linear scale and the right graphs present the same data on a semi-log scale.

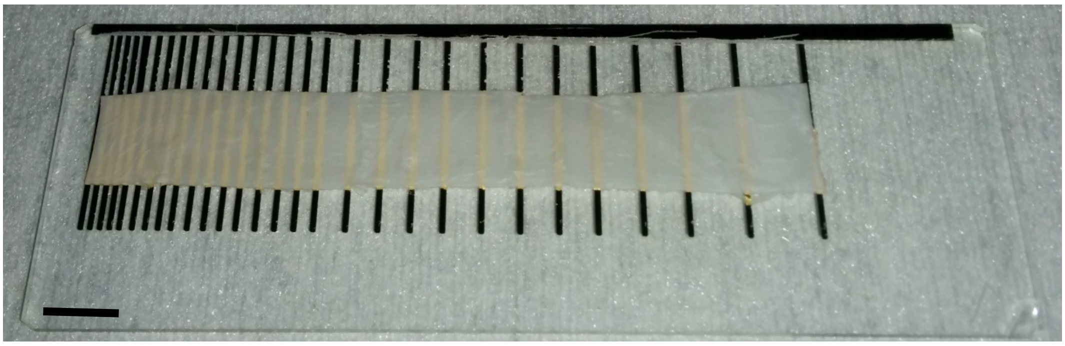

Figure S7. Image of the BSA mat on the gold finger array electrode. The bar represents 5 mm. The Au fingers are not connected to the Au bar in the top of the image, and they are separated from each other.

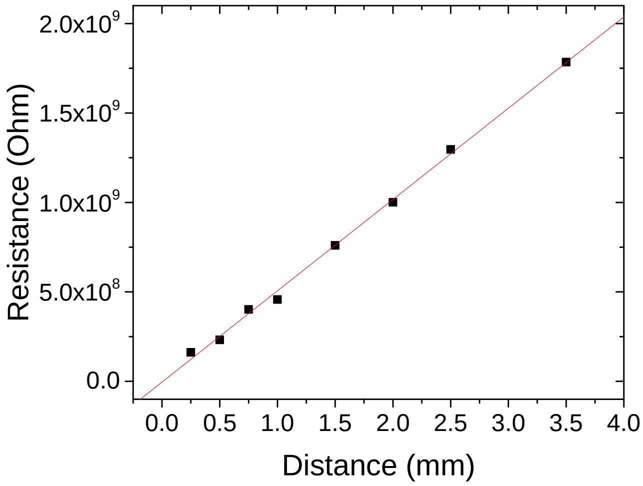

Figure S8. Contact resistance estimation for the BSA mats. The contact resistance was estimated by extrapolation of the resistance to a hypothetical electrode separation distance $l = 0$. The deduced contact resistance is negligible for the conductance measurement across the mat (since the resistance across the mat is significantly higher).

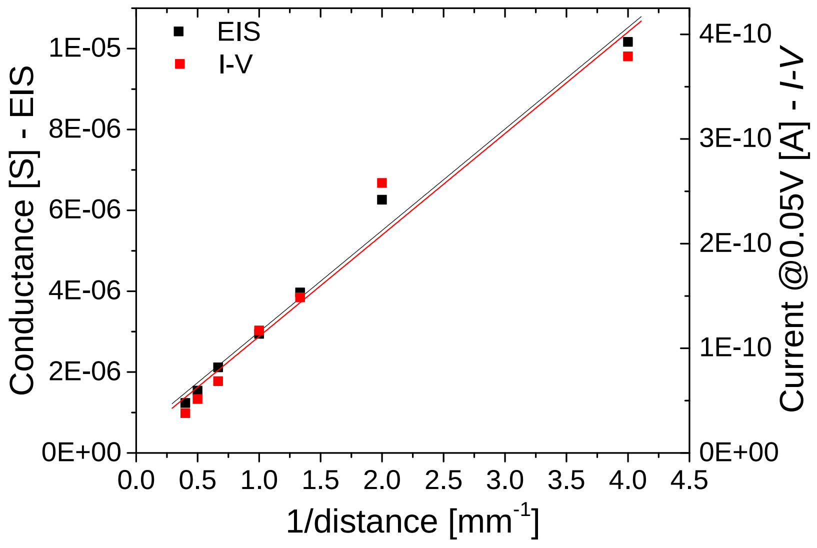

Figure S9. The EIS conductance (left y-axis, black squares) and *I-V* current at 0.05V (right y-axis, red squares) as a function of 1/distance.

Table S2. Resistance and capacitance values as measured by EIS and fitted to the below electric circuit.

Distance [mm]	Calculated Resistance [Ω]	Calculated Capacitance [F]	Conductivity [S·cm^{-1}]	Electric Circuit
**0.25**	0.98±0.30·10^{5}	4.32·10^{-12}	4.11·10^{-5}	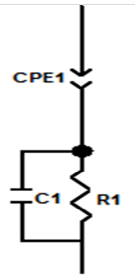
**0.5**	1.60±0.39·10^{5}	3.68·10^{-12}	4.88·10^{-5}	
**0.75**	2.52±0.98·10^{5}	2.98·10^{-12}	4.65·10^{-5}	
**1**	3.39±1.35·10^{5}	2.33·10^{-12}	4.63·10^{-5}	
**1.5**	4.72±1.81·10^{5}	1.50·10^{-12}	5.01·10^{-5}	
**2**	6.49±2.73·10^{5}	1.28·10^{-12}	4.86·10^{-5}	
**2.5**	8.08±3.04·10^{5}	1.14·10^{-12}	4.86·10^{-5}	

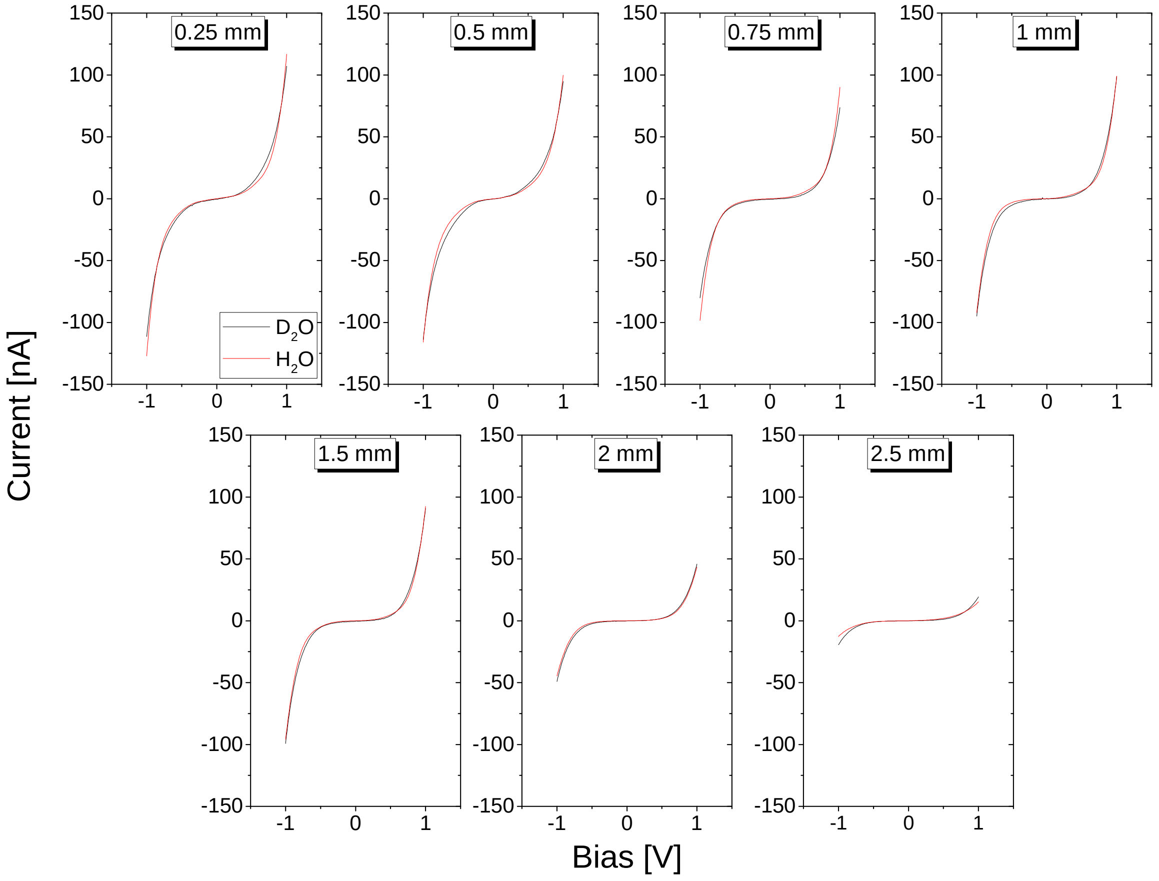

Figure S10. *I-V* curve KIE data for all of the measured inter-electrode separation distances.

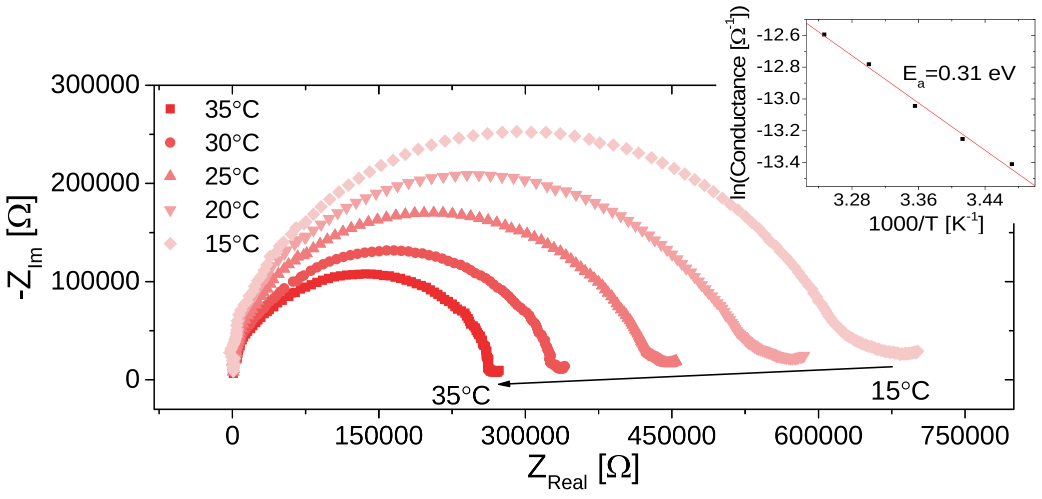

Figure S11. Temperature dependence of the EIS for a 1.5 mm inter-electrode separation distance. The inset shows the activation energy of the process by fitting to an Arrhenius equation.

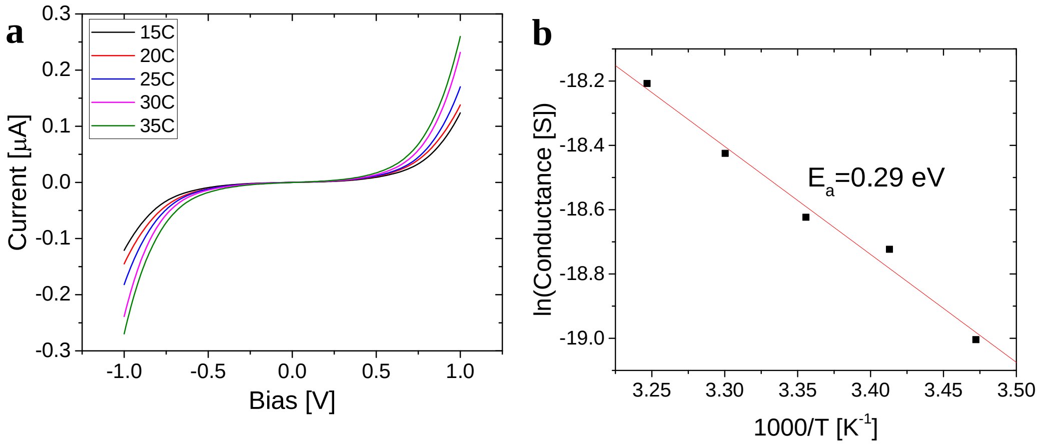

Figure S12. (a) Temperature dependence of *I*-*V* data for a 0.75 mm inter-electrode separation distance. (b) The carrier transport activation energy (~0.29 eV) was estimated by an Arrhenius fit (red line) to the extracted conductance data (filled squares).

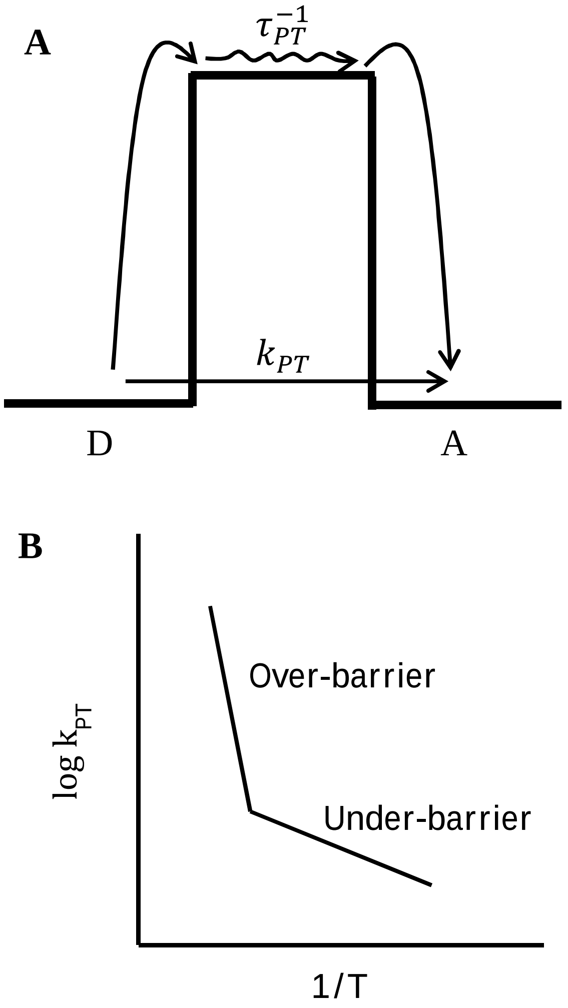

Figure S13. (a) Schematic representation for proton transfer mechanisms from proton donor (D) to acceptor (A). The bottom straight arrow represents *under the barrier* proton transfer, characterized by a large proton transfer rate (k_{PT}) in the order of 0.3-1×10^{12}. The curved arrows represent the trajectory of *over the barrier* proton transfer. In the latter case the proton interacts with the barrier and resides thereon, resulting in a much longer proton lifetime (τ_{PT}).

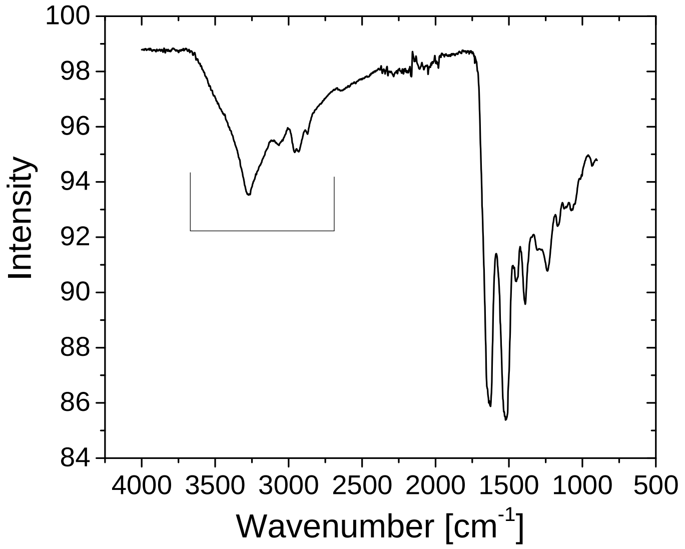

Figure S14. FTIR spectrum of a dry BSA mat. The inset bracket identifies vibrational modes associated with charged amino acids. The peaks around 1640 and 1530 cm^{-1} correspond to amide I (C═O stretching) and amide II (C–N stretching, N–H bending) moities respectively.

Supplemental References

[1] S. Fleischer, A. Shapira, O. Regev, N. Nseir, E. Zussman, T. Dvir, *Biotechnol. Bioeng.* **2014**, *111*, 1246-1257.

[2] E. Pines, D. Huppert, N. Agmon, *J. Chem. Phys.* **1988**, *88*, 5620-5630.

[3] N. Agmon, E. Pines, D. Huppert, *J. Chem. Phys.* **1988**, *88*, 5631-5638.
